# Growth functions of periodic space tessellations

**DOI:** 10.1107/S2053273324010763

**Published:** 2025-01-01

**Authors:** Bartosz Naskręcki, Jakub Malinowski, Zbigniew Dauter, Mariusz Jaskolski

**Affiliations:** a Faculty of Mathematics and Computer Science, Adam Mickiewicz University, Poznań, Poland; b Mathematical Institute Polish Academy of Sciences Warszawa Poland; c Faculty of Pure and Applied Mathematics, Wrocław University of Science and Technology, Wrocław, Poland; d Macromolecular Crystallography Laboratory NCI Argonne National Laboratory Argonne USA; e Department of Crystallography, Faculty of Chemistry, Adam Mickiewicz University, Poznań, Poland; f Institute of Bioorganic Chemistry Polish Academy of Sciences Poznań Poland; Institute of Crystallography - CNR, Bari, Italy

**Keywords:** tiling of the plane, tessellations, modified Euler characteristic, orphic diagrams, topological growth functions, crystallographic growth functions

## Abstract

This work analyzes the rules governing the growth of the numbers of vertices, edges and faces in all possible periodic tessellations of the 2D Euclidean space, and encodes those rules in several types of polynomial growth functions. These encodings map the geometric, combinatorial and topological properties of the tessellations into sets of integer coefficients. Several general statements about these encodings are given with rigorous mathematical proof. The variation of the growth functions is represented graphically and analyzed in orphic diagrams, so named because of their similarity to orphic art. Several examples of 3D space groups are included, to emphasize the complexity of the growth functions in higher dimensions. A freely available Python library is presented to facilitate the discovery of the growth functions and the generation of orphic diagrams.

## Abstract 2

For any periodic tilings of the plane by congruent polygons, growth functions are derived that count the numbers of vertices, edges and faces as the coverage expands. The functions are computed as polynomial formulas in a Python program and analyzed graphically using orphic diagrams.

## 1. Introduction

**Figure 1:**
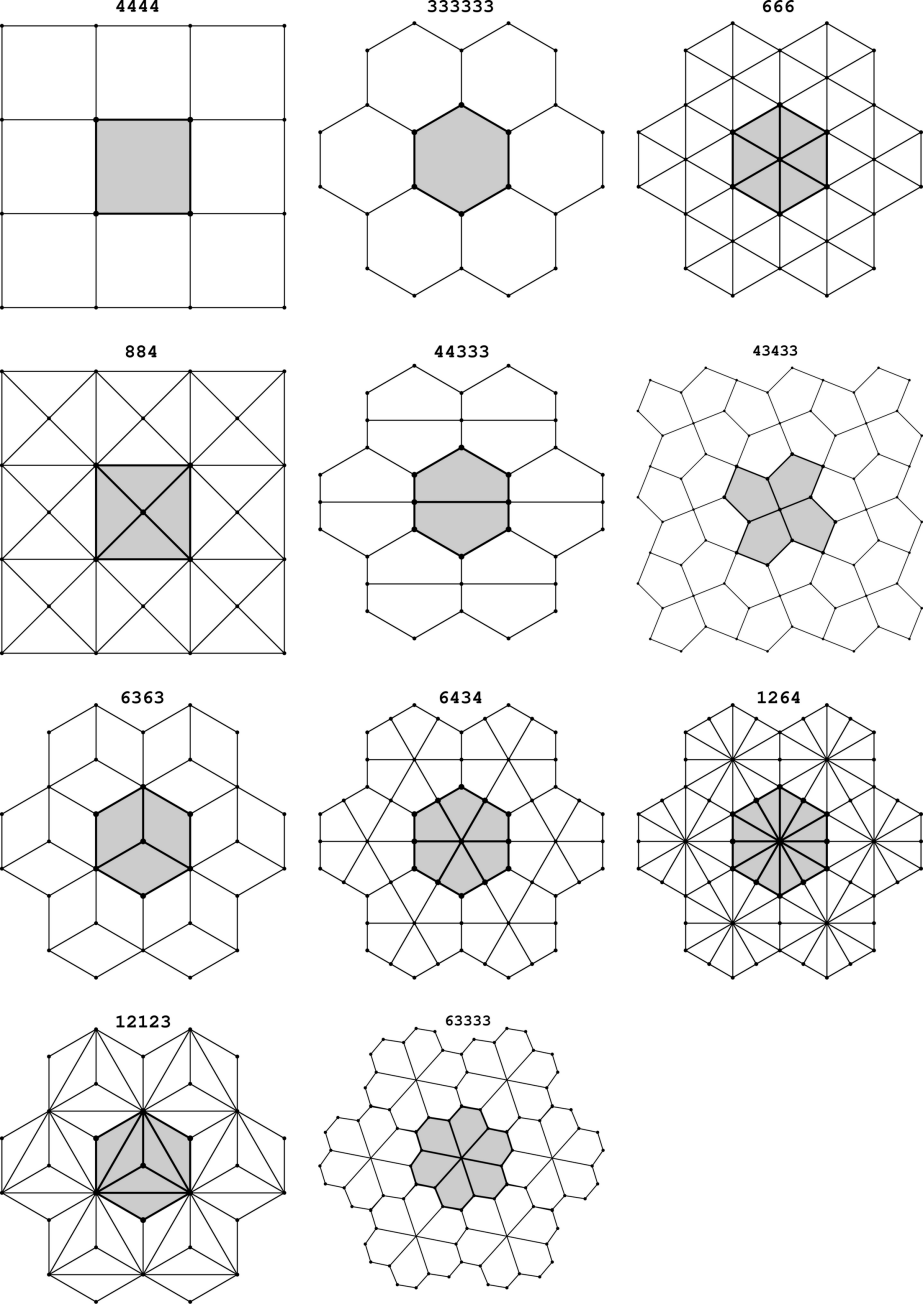
Eleven topologically distinct types of planar tilings in their most symmetric representation. Each type is characterized by the vertex valences and by the space-group symmetry (in parentheses). 4444 (*p*4*mm*), 333333 (*p*6*mm*), 666 (*p*6*mm*), 884 (*p*4*mm*), 44333 (*c*2*mm*), 43433 (*p*4*gm*), 6363 (*p*6*mm*), 6434 (*p*6*mm*), 1264 (*p*6*mm*), 12123 (*p*6*mm*), 63333 (*p*6).

Our focus in this paper is on periodic tessellations of space. In 2D there are 11 possible, topologically distinct, periodic tilings by congruent polygons, also known as Laves tilings (see Appendix *A*[App appa], where some terms are explained in the glossary). These tilings are designated by listing the numbers of edges that meet at each of the vertices (the number of edges meeting at a vertex is called its valence) of the tile (*cf*. Fig. 1 ▸). For example, 4444 denotes a grid created by two sets of parallel lines. The unit cell of such a grid is a parallelogram. In the familiar case of crystallographic tessellation, the space is covered by a periodic (lattice) arrangement of identical unit cells. The unit cells are in turn subdivided, according to space-group symmetry, into asymmetric units (ASUs), which can be selected in many ways, but cannot be subdivided further without loss of symmetry. We start from a lattice node in 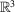 and expand our frame of coverage, which is congruent to the crystallographic unit cell, and count the number of interiors (I) covered. That number grows in a cubic fashion, as do the counts of other geometrical elements, such as vertices (V), edges (E) and faces (F). In the general Euclidean space 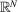, the same polynomial type of count holds for any *k*-dimensional cell (Appendix *A*[App appa]) for *k* between 0 and *N*. In the case of 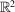, the polynomials are quadratic functions. But what exactly are these functions? Do they depend on the choice of the origin, or the choice of ASU, or on the frame that we expand to cover a growing area of the lattice? Are there rules governing their form? These and other questions were at the root of our interest in the subject of growth functions. In our previous work (Dauter & Jaskolski, 2020 ▸; Naskręcki *et al.*, 2021*a* ▸, 2021*b* ▸, 2022 ▸) we investigated the general asymptotic behavior of the growth functions. In the current paper we present, with proof, the exact formulas for the polynomial growth functions of the permitted periodic tessellations in the 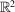 space.

We also probe the possibility of using the growth functions to expose the underlying lattice when its regularity is distorted by, *e.g.*, lattice defects; that particular application of the growth functions might be of practical utility (*cf*. Section 7.2). Finally, we remark that a similar problem of tessellating the 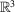 space by parallelohedra would be dramatically more complicated.

## 2. Panorama of the ideas

**Figure 2:**
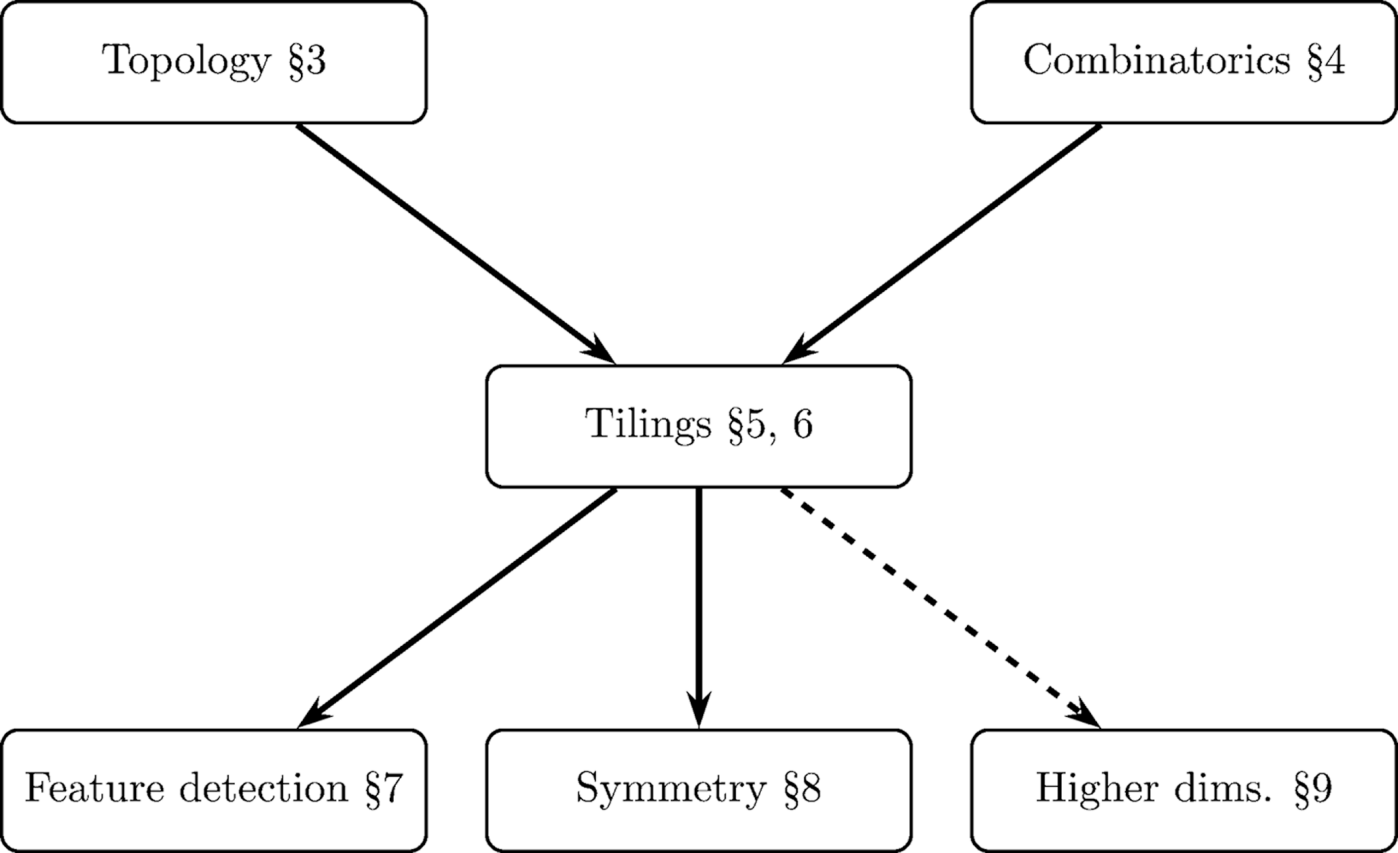
Flow of ideas throughout the paper.

To aid the readers in navigation through the text, we describe in this section the main ideas of our work and illustrate their flow in the paper in Fig. 2 ▸.

*Topology*. Our point of departure is an idea from topology to treat the process of tessellating the space 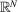 as one in which we start from a single point (0-cell or vertex) and attach to it certain *N*-dimensional cells. The collection of cells (with the information about how the edges, faces *etc*. are connected) will constitute a fundamental topological structure of our objects. Our principal requirement is that these *N*-cells, taken together, must satisfy the property that under shifts in space (by integral multiples of *N* linearly independent vectors) they give an exhaustive tessellation of the space, or the so-called periodic tessellation.

*Combinatorics*. Our major goal is to encode topological and combinatorial data in the form of functions that count, for a fixed *k*, the number of *k*-cells of the tessellation within the bounded number of copies of the fundamental region (Appendix *A*[App appa]). We will discuss in detail two very different approaches to this counting procedure. One, which we call topological, relies only on the combinatorial data, *i.e.* the count of connected cells *etc*. The other way, which we call crystallographic, will count the cells with respect to their position in a growing parallelepiped rooted in various points. The crystallographic version is very sensitive to shifts, hence we encode those functions in the form of a color diagram, called an orphic diagram (Appendix *A*[App appa]).

*Symmetry*. A major reason to look at the above two variants is that the former one is ‘crude’, *i.e.* it is insensitive to the change of the symmetry group of a tessellation when the topological type is preserved. For a simple example think about a tiling of the plane by squares and a tiling by general parallelograms. Topologically they are identical but they differ at a subtler level of geometric deformations.

*Tilings*. We build a complete set of formulas for the topological growth functions of 2D tilings. This idea is then enhanced by using a certain modification of the topological growth function construction. We change our counting procedure by making a reference to the expanding unit cell. These so-called crystallographic growth functions can be visualized by color diagrams.

The classification of the possible topological growth functions is important for the more refined crystallographic approach. Many properties of these functions are shared but, as we illustrate in several examples, the crystallographic growth functions have the potential to completely encode the symmetry group, *i.e.* two isometric tessellations will have similar growth function diagrams.

*Feature detection*. We discuss certain simple applications of this encoding of the properties of the tilings and are laying the ground for space-group symmetry recognition in future projects.

*Higher dimensions*. Finally, we discuss the prospects of generalizing our results to higher dimensions. Serious obstacles arise both conceptually and practically. In principle, the tools that we have developed are easy to apply to any particular tessellation in higher dimension but building a conceptual description of the growth functions in higher dimensions is more challenging.

## 3. Tessellations

We denote by 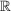 the set of real numbers and by 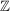 the set of integers contained in 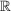. Let 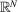 denote the *N*-dimensional Euclidean space, *i.e.* the set of *N*-tuples (Appendix *A*[App appa]) 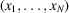 of real numbers 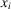. Let 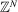 denote the set of *N*-tuples 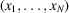 of integers 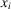. By construction, the set 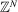 is properly contained in 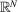. (Notice that the set of integers 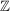 is countable, while the set of real numbers 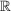 is uncountable.)

In this paper we deal with the notion of a tessellation of the Euclidean space. We discuss in this section the necessary mathematical preliminaries from geometry, topology and combinatorics that will be used throughout the paper. The different notions of equivalence between tessellations are explained by Grünbaum & Shephard (1987 ▸, Sections 1.3, 4.1).

### 3.1. Normal tessellations

In this paper we work with the so-called normal tessellations in the sense of Grünbaum & Shephard (1987 ▸, Section, 3.2). This definition can be applied to tessellations in an arbitrary number of dimension of the Euclidean space 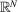. In the sense given above, a tessellation is a countable family of closed sets 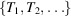 which cover the space without ‘gaps’ or ‘overlaps’. The lack of gaps means that the union 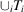 of the covering sets gives us 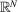. The lack of overlaps means that the interiors of 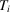 and 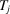 for any 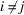 do not overlap, *i.e.* the sets 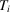, 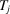 can meet only on the boundary (*cf*. Grünbaum & Shephard, 1987 ▸, Section 1.1). The tessellation is normal if:

(i) Every set 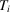 is a topological *N*-dimensional ball, *i.e.* up to bi-continuous bijection (homeomorphism) every set 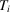 is an *N*-dimensional ball.

(ii) The intersection of every two sets 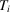, 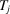 is a connected set.

(iii) The sets 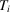 are uniformly bounded, *i.e.* there exist two radii *r* and *R* such that every set 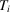 contains a ball of radius *r* and is contained in a ball of radius *R*.

Conditions (i)–(iii) represent for us the most interesting and easy to understand ways of tessellating a space. In particular, all regular tessellations, *e.g.* the crystallographic ones, are contained in this class. Notice also that it follows from (i) and (ii) that the intersection of every two different 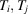 consists of a single ‘part’. Conditions (i)–(iii) imply that the tessellation of space with 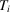 ‘grows’ moderately (Grünbaum & Shephard, 1987 ▸, Section 3.2).

### 3.2. Periodic tessellations

Our focus in this paper is exclusively on *periodic* normal tessellations.

Let 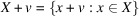 be a shift of the set 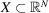 by a vector *v*. We denote by 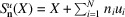 a shift of *X* by the linear combination of vectors from 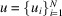 indexed by 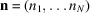.

Definition 3.1 We say that a normal tessellation 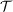 (of 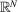) is periodic if there exists a covering family 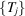 and *N* linearly independent vectors 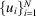 such that, for any 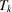, 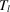 there exists an *N*-tuple of integers 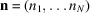 for which 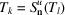
holds. Since all sets 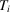 are equivalent by integral shifts, we fix one of them, say 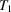, and denote it Ω. We call Ω a *generating region* of 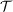.

For every two distinct vectors 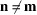, the shifts 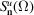, 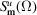 overlap possibly on the boundaries. We denote by 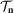 a CW complex (Appendix *A*[App appa]) which is the union of CW complexes 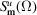 where 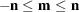. Every complex 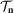 is finite and satisfies 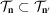 for every 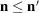 (the inequality relation is lexicographic). We call the family 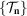 indexed by *N*-tuples 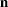 a stratification of 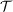.

*Remark.* A periodic tessellation can be constructed with a set Ω that is not necessarily a polygon (curvy edges). This would be rather unusual in crystallography but can be applied, for example, to the tilings of M. C. Escher *etc*.

### 3.3. CW structure

Tessellation in this paper is considered as a topological object, *i.e.* a CW complex (Appendix *A*[App appa]). We refer the reader to our mathematical glossary for a fuller description. Also, more details about CW complexes can be found in standard textbooks (Whitehead, 1949 ▸; Hatcher, 2002 ▸). In brief, a CW complex is a topological space with extra data about how it was obtained from gluing simple pieces which are called cells. Every cell is topologically a ball of appropriate dimension. A tessellation of the space 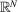 is obtained by gluing together *k*-dimensional cells of dimensions ranging between 0 and *N*.

We start from a simple example. Consider a closed interval 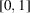 on the real line 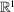. The CW structure on 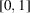 can be determined by the following procedure. We have two 0-dimensional cells, *i.e.* the vertices 0 and 1. We connect them by (glue to them) a closed interval 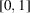, where the end point 0 of the interval matches vertex 0 and the end point 1 matches vertex 1. There are no other cells in this CW structure. The sets of *k*-dimensional cells will be denoted 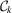.

In particular, for a tessellation in 2D, which we call a tiling, we write 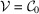 for the set of vertices, 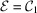 for the set of edges and 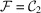 for the set of faces. The sets 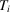 in this context are called tiles.

Example 3.2 Addition of the connectivity structure (CW structure) of a tessellation allows one to define objects such as paths and count properly the objects in the tessellation (such as faces or edges) that are similar. Consider the real line 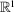
as a union of closed intervals 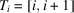 for 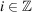. The vertices which connect the segments 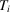 are the integers 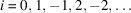. Notice that the endpoints of the intervals 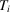 overlap between neighboring segments. The edges are represented by the segments 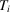. Every point on the real line is contained either in a single segment (interior of the edge) or belongs to finitely many (in this case exactly two) segments; these are the points from the vertex set.

### 3.4. Vertex notation

Any monohedral tiling (*i.e.* having exactly one prototile, where a prototile in a tiling is a face which has no sub-faces) with tiles whose vertices (ordered cyclically) have valences (*i.e.* numbers of meeting edges) 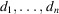 will be denoted 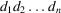. In our limited context, the numbers 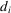 belong to the set 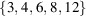, hence no confusion arises when we write a sequence like 12123, which stands for 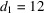, 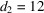 and 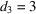. This is a simplified version of the notation introduced by Grunbaum & Shepherd (1987 ▸, Section 2.7).

## 4. Growth functions

In this section we introduce a certain topological and geometric tool that will be used to study tessellations, the growth functions. One can define such functions in various ways, depending on the context.

The growth functions that we introduce in this paper fit into a more general framework of mathematical objects that can be attached to tessellations. Our main motivation came from our previous work, where we investigated a variant of the classical Euler characteristic (Appendix *A*[App appa]), which could be adopted for the notion of crystallographic tessellations. The connection makes use of the orbifold (Appendix *A*[App appa]) variants of the Betti numbers used in topology to describe the number of ‘holes’ in the topological space. In fact, the Betti numbers are in correspondence with the leading coefficients of our topological growth functions, provided that the structure of the associated orbifold is simple (Naskręcki *et al.*, 2021*a* ▸).

For general tilings, previous authors studied the combinatorics (counting the objects) of the tessellations using various tools like graphs and generating functions. The latter are especially suited and useful for studying tessellations which are non-periodic because in these situations a finite sequence of numbers cannot in general determine the structure of such tessellations. [Compare this with the famous Penrose tilings. The ways in which one can build such a tiling can be identified with the whole uncountable set of real numbers. This means that a finite amount of data is usually not enough to characterize such tilings (Senechal, 1995 ▸, Section 6.2).]

We also notice some similarity with the work of Grosse-Kunstleve *et al.* (1996 ▸) on coordination sequences for zeolites. These authors build certain growth functions associated with zeolites and study their properties. In their approach the counting is done by introducing a shelling of the tiling.

### 4.1. Topological/combinatorial approach

Definition 4.1 (Topological growth function) For a normal periodic tessellation 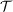 of 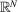 with generating region Ω and with the finite stratification 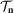 of the Euclidean space 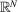 we define for every integer *k* in the range between 0 and *N* a function 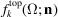 where 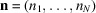 is a coordinate vector of non-negative integers. The function 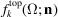 counts the number of *k*-cells in the finite CW complex 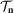.

It follows from Naskręcki *et al.* (2021*a* ▸) that the functions 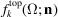 are polynomials of total degree *N* with integer coefficients.

For our particular Example 3.2[Statement example3.2], starting from any vertex (integer) *m* we obtain functions 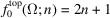 and 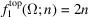, where 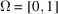.

Example 4.2 Tiling 4444 – a mathematical perspective. The plane 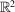 can be given a CW structure by considering a union of unit squares (with boundary) which tiles the space. This CW structure has the following description:

(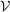) The set of vertices is the set of pairs 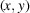 with integer coordinates 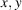, *i.e.*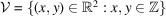.

(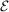) Edges are segments 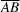 of length 1 [or ordered tuples 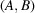] which span the space between two distinct points 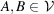, *i.e.*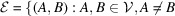}.

(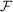) Faces are unit squares with vertices in the set 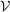. One can identify such a face with an ordered tuple 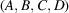 of elements 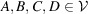. Let *U* denote the quadruple 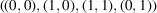. Under a suitable translation vector 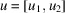 with integer coordinates every face 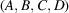 satisfies 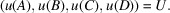
Hence we define the set of quadruples 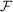 = 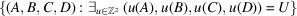.

For every face 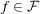 we define the geometric square, *i.e.* a subset of 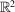 which corresponds to *f*. The totality of such squares provides a tiling 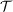 of the space 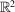 with a generating region 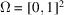.

For every vertex *v* and fixed *k*, the functions 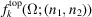 are the same. In fact 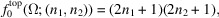

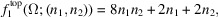

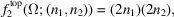
where 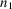, 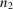 are the number of positive and negative steps in both directions of the 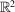 space.

*Note*. In the following (except Section 5), each time we write a growth function we apply a standard normalization, *i.e.* instead of growth in each direction we only consider the growth in the ‘positive’ directions. Hence the normalized forms of the latter functions would be 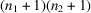, 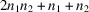 and 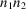, respectively.

### 4.2. Crystallographic approach

In our considerations we limit ourselves to the choice where the index set is the set of non-negative integers and we measure the growth of the tessellation 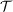 against the propagation of the unit cell, which is a parallelogram (or in general an *N*-parallelepiped), with a fixed starting point.

For a periodic tessellation with generating period vectors 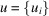, we define an *N*-parallelepiped 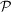 = 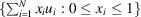 spanned by the vectors in *u*. Now, we have a family of growth functions (indexed by dimension *k* and starting point 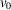) which for a given non-negative integer vector 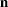 count the number of *k*-cells of 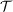 which are completely contained in the set 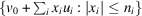. We call such functions ‘crystallographic’ growth functions, to distinguish them from the previously defined (for not necessarily periodic tessellations) more ‘topological’ growth functions.

Let us formalize now the notion of crystallographic growth functions.

Definition 4.3 (Crystallographic growth function) Let 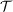 be a periodic normal tessellation of 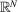 with generating region Ω and translation vectors 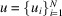. Let 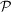 denote the parallelepiped spanned by *u*.

We denote by 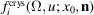 the number of *i*-dimensional cells of the tessellation 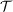 which are completely contained in the region 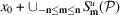. Similarly, we define 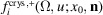 as the function which counts the *i*-dimensional cells in 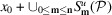.

We denote by 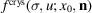 the number of *u* translates of the cell 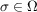 of the tessellation 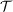 which are completely contained in the region 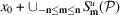. Similarly, we define 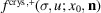 as the function which counts the translate of σ in 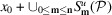.

The crystallographic growth functions have a more interesting behavior, in particular they are sensitive to the choice of the starting point 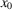 in 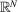. Their behavior in 2D is discussed in detail in Section 6.

### 4.3. Mantissa equations

We show in this section that the crystallographic functions are the same in certain ‘small’ regions. More precisely, there exists a tessellation of 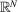 with the rescaled version of the parallelepiped 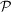 spanned by the period vectors *u*. Starting from a point 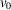 we attach to it a scaled parallelepiped 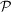 and proceed accordingly further. The choice of scalings arises from the following simple observation.

Let 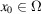 be a point and let 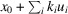 be a shifted point, where 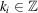. The parallelepiped 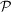 generates via the periodic translations a tessellation. It follows that 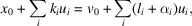
where 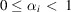, 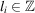. Hence, we have 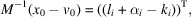
where *M* is a matrix 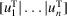 formed from the vectors in the set *u*. It is invertible since 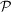 has non-zero volume. We let ω denote the vector 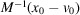 with elements 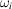. Let 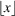 denote the integer 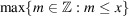. Since 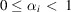, it follows that for each *i* we have the following mantissa equations:
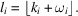
The region in 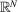 for which 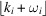 is fixed is a parallelepiped. This condition determines for which values 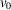 a growth function change (‘jump’) occurs (*cf*. Section 6).

We can summarize the properties of the crystallographic growth functions in the following.

Lemma 4.4 Let 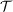 be a normal periodic tessellation of 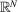 with generating region Ω and translation vectors *u*, spanning the parallelepiped 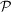. The following hold:

(i) For any two points 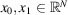 such that 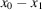 is an integral linear combination of the vectors in *u*, the *N*-tuple of functions 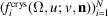 is the same for 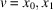.

(ii) For any 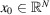 and any *i*, the function 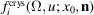 is for a sufficiently large 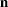 a polynomial in variables 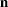 of total degree *N* with integer coefficients.

**Proof**

Property (i) follows from the mantissa equations discussed above. Proof of (ii) follows from the analogous calculations for the topological growth functions (*cf*. Naskręcki *et al.*, 2021*a* ▸).

□

Now we want to prove that the set 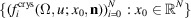
of vectors of crystallographic functions is finite. We decompose each function 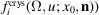 into a sum of constituents which count only the number of appearances of a single cell. We will show that for each cell the growth functions are particularly simple and explicit.

Let 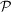 be a parallelepiped generated by the vectors 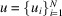. Let 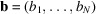 be a vector of any real numbers, including zero. Let 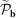 denote the set of vectors 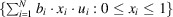. We call 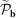 a dilate of 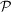.

We prove below that we have a periodic normal tessellation of 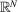 with dilates of 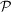 such that on every interior of any *k*-dimensional cell the *N*-tuple of crystallographic growth functions remains fixed.

Definition 4.5 We call a periodic normal tessellation 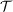 with translation vectors *u* a *u* tessellation if all cells are parallelepipeds generated by 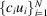 for some non-negative numbers 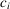.

For any cell 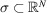, we define:


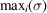
 = 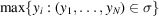,


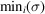
 = 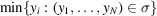.

Here, 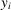 denotes the *i*th coordinate of the point 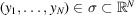. In other words, we are taking the maximum or minimum value of the *i*th coordinate across all points within the cell σ.

Definition 4.6 Let 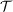 be a periodic normal tessellation with generating region Ω and translation vectors *u*, and spanning the parallelepiped 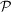. Let 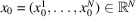 be fixed. We say that a cell 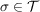 appears after 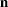 = 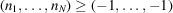 extensions with respect to 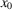 if for 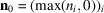:

(i) 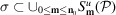 and 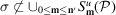 for any 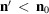;

(ii) We have 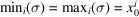 exactly for those *i* where 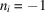.

We say that 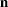 is the order of appearance of σ with respect to 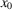.

Lemma 4.7 Let 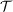 be a periodic normal tessellation with generating region Ω and translation vectors *u*. Let 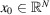 be fixed. Let σ be a cell in Ω. Let 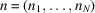 be the order of appearance of σ with respect to 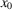. It follows that for 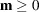 we have 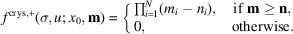

**Proof**

We note that for two subsets 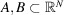 any linear transformation 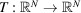 that transforms the basis *u*into an orthonormal basis 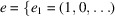, 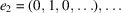, 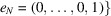 satisfies the property 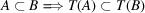. The definition of 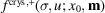 is based purely on inclusions of sets, hence we can assume without loss of generality that 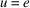. Let us denote in this proof the function 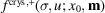 by 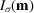. We will first prove that 
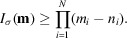
Let 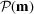 be the parallelepiped 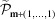. Let 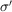 be a certain *u*-integral translate of σ appearing after 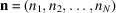 extensions. We assume that 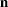 is coordinatewise minimal. Let 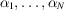 and 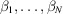 be natural numbers such that for all *i* we have inequalities 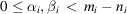. It follows that 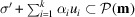 and 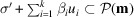 but if for any *i* we have 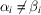, then 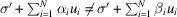. So the following inequality holds true:
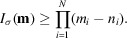

We will now demonstrate the inequality in the other direction. Without loss of generality, we can assume that 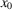 is at the origin of the coordinate system. To prove the inequality by contradiction, let us suppose that 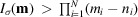. From this assumption, it follows that there exists a cell 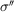 which is a *u*-integral translate of σ and is contained in 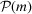 such that if for all *i* we have 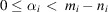, then 
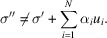
On the other hand, 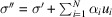 for some integral vector 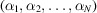. Hence, there exists *i* such that 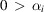 or 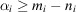.

Case: 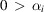. If 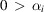, then the *i*th coordinates of vertices of 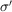 are bigger than 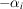. So 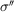 is contained in 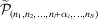. Note that 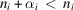. So the cell σ appears inside the frame after 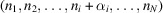 extensions, a contradiction with the assumption.

Case: 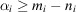. If 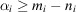, then the *i*th coordinates of vertices of 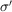 are less than or equal to 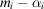, which is not larger than 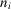. Thus, the cell σ appears already after 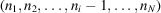 extensions of the frame, a contradiction with our assumption.

□

Now we want to compute a formula for the order of appearance of any cell σ with respect to 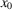, 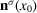. For every 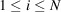, let 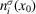 denote the *i*th coordinate of the vector 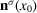.

For any set *A*, we denote by 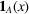 the function that is equal to 1 for all 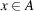 and 0 otherwise. We denote by 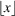 the number 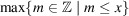 and by 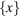 the number 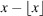.

Lemma 4.8 Let 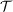 be a periodic normal tessellation with generating region Ω and translation vectors 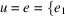 = 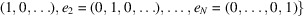. Let 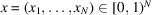 be fixed. Let σ be a cell in Ω. For every 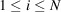 we have 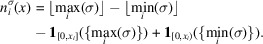

**Proof**

The proof is independent and essentially identical for each fixed coordinate index *i*.

Let us fix *i*. The projection map 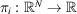 is an open mapping, hence the image 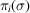 is an interval 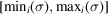. The rank of appearance of σ in direction *i* with respect to 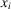 equals 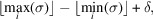
where δ depends on the location of the numbers 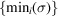,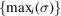 in the unit interval. We have

(i) δ = −1 when 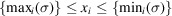;

(ii) δ = 0 when 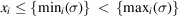 or 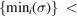

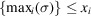
 or 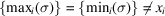;

(iii) δ = 1 when 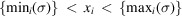.

□

Theorem 4.9 Let 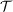 be a periodic normal tessellation with generating region Ω and translation vectors *u*. Let σ be a cell in Ω. There exists a *u* tessellation 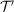 such that the function 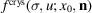 of variable 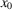 remains constant in the interior of every *k*-dimensional cell of the CW structure induced on 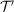.

**Proof**

Since the function 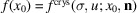 is a sum of finitely many functions of type 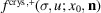, we just need to prove that such an invariant *u* tessellation exists for 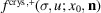.

Without loss of generality we can assume that 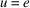, the orthonormal standard basis. Indeed, it follows from Lemma 4.8[Statement lemma4.8] and Lemma 4.7[Statement lemma4.7] that the function 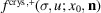 has an invariant *u* tessellation which consists of 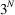 parallelepipeds (possibly degenerate). In each coordinate direction the unit cell is divided into three segments, following the step function defined in Lemma 4.7[Statement lemma4.7].

□

Theorem 4.10 Let 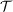 be a periodic normal tessellation with generating region Ω and translation vectors *u*. Let 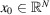 be fixed. Let 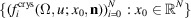
be the set of crystallographic growth functions attached to 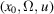. There exists a *u* tessellation 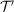 such that, for every *i*, the function 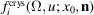 of variable 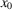 remains constant in the interior of every *i*-dimensional cell of the CW structure induced on 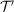.

**Proof**

For each *i* the function 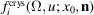 is a sum of the functions 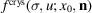, where summation ranges over *i*-dimensional cells σ of Ω. In fact, the summation goes over the equivalence classes of cells up to translation relation.

It follows from Theorem 4.9[Statement theorem4.9] that each function 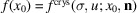 has a *u* tessellation 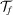 for which the function 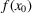 remains fixed on the interiors of each cell. To conclude, we need the following claim.

*Claim*. When given two such functions 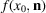 and 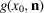, with respective *u* tessellations 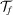 and 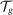 on which the functions *f* and *g* are constant (with respect to 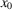) on the interiors of cells, the function 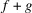 has a corresponding *u* tessellation 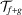 with the same constancy property as *f* and *g*. The new tessellation is obtained from the common subdivision of 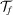 and 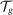.

*Proof of the claim*. To prove the Claim, it is enough to notice that an intersection of two parallelepipeds generated by rescales of *u* is again a parallelepiped.

□

Since the function 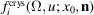 is a sum of 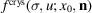 over finitely many cells σ, the claim proves our theorem.

□

The properties of the crystallographic growth functions, including their pictorial representations in 2D, are discussed in Section 6. A simple, yet non-trivial example of this setup is illustrated by the data in Fig. 11 and the dilated regions are shown in Fig. 5(*a*). Interested readers can check the actual ratios by executing our code (Malinowski, 2022 ▸) and inspecting the outputs.

Corollary 4.11 Let 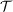 be a periodic normal tessellation with generating region Ω and translation vectors *u*. The set of *N*-tuples of polynomials 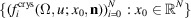
is finite.

**Proof**

Let *u* denote the translation vectors associated with 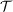 and Ω. Let 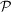 be a parallelepiped spanned by *u*.

Because of Theorem 4.9[Statement theorem4.9], there exists a normal periodic tessellation 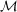 with dilates of 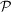 such that the vector 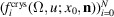 remains fixed for every 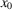 in the interior of every cell in 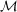. Due to the periodic character of the tessellation and to the finite, up to translations in *u*, CW structure on the tessellation, we obtain the final conclusion.

□

Definition 4.12 Let 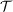 be a periodic normal tessellation with generating region Ω, translation vectors *u* and associated parallelepiped 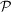. Due to Corollary 4.11[Statement corollary4.11] there exists a tessellation 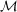 of 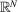 built out of dilates of 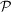 such that we have a function 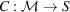 to a certain finite set *S*. The function *C* has the following properties:

(i) The elements of the set *S* are in bijection with the set 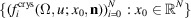.

(ii) For every element 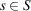 the preimage 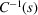 is the set of points 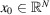 for which the vector 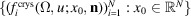 remains fixed.

(iii) For every closed cell σ in 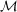 the function *C* is closed on the interior of the cell σ.

We call 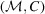 the ‘crystallographic coloring’ of the space 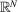 with respect to the quadruple 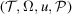.

The beautiful pictorial representations of the crystallographic colorings are discussed further and shown in Section 6.

## 5. Topological growth functions of a tiling

Starting from the current section until the end of Section 8, we restrict our attention to the case of normal periodic tessellation of the plane. Such a tessellation 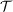 will be called a *tiling* and the fundamental domain which covers the plane by translations will be called a tile and denoted Ω. (In this context, the tile is the unit repeated only by translation; note that in the crystallographic context it may be further divided into smaller tiles, possibly related by other symmetry operations.) We consider Ω and the tiling 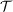 with their CW structure. The reason for the restriction to dimension 2 only is twofold. First, we have a complete description of the topological growth functions in terms of the combinatorial data attached to Ω, so far, only in dimension 2 (*cf*. Theorem 5.5[Statement theorem5.5]). Second, in Section 6 we develop the theory of crystallographic growth functions of the tilings. This theory can be developed in higher dimensions as well but visualizations of the growth functions become very cumbersome in higher dimensions.

Assume we have a closed, simply connected CW complex 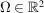 and two linearly independent vectors 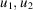 such that the shifts of Ω by 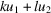 for any 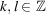 meet only at the boundaries and give rise to a normal periodic tiling of the plane. We require that the vertices are translated to other vertices and edges to edges. We fix a certain 0-cell (a vertex) 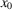 which we call the origin of the tiling 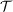. Let 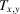 denote a tile which is 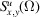. We denote by 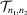 the CW complex obtained as the union of all tiles 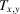 with 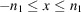 and 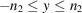. We denote by 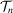 the CW complex which is the union of tiles 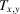 with 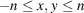. We are going to describe the formulas 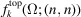 for the number of *k*-dimensional cells in 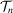. We will generalize this also to the growth function for 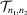. It turns out that the only data required to describe these functions are the combinatorial data attached to Ω, *i.e.* the number of vertices, edges and faces.

It is a consequence of the Laves classification (Grunbaum & Shepherd, 1987 ▸, Section 2.7; Girault-Beauquier & Nivat, 1991 ▸) that the boundary of Ω can be divided into six connected 1-complexes (two possibly degenerating to a single point) such that among them there are three pairs of congruent 1-complexes, *i.e.* unions of edges. The two model cases are a parallelogram and a hexagon. Topologically speaking, the parallelogram case corresponds to a standard construction of a topological torus with the identification of the translation-equivalent opposite pairs of edges. The hexagon case under the identification of the edges in the first pair leads to a tube with two boundary circles oriented in the ‘wrong way’. By twisting the tube by 180° (via imaginary topological willpower), one can correctly identify the missing two pairs, obtaining yet again a topological torus but with a non-trivial twisting along the tube.

**Figure 3:**
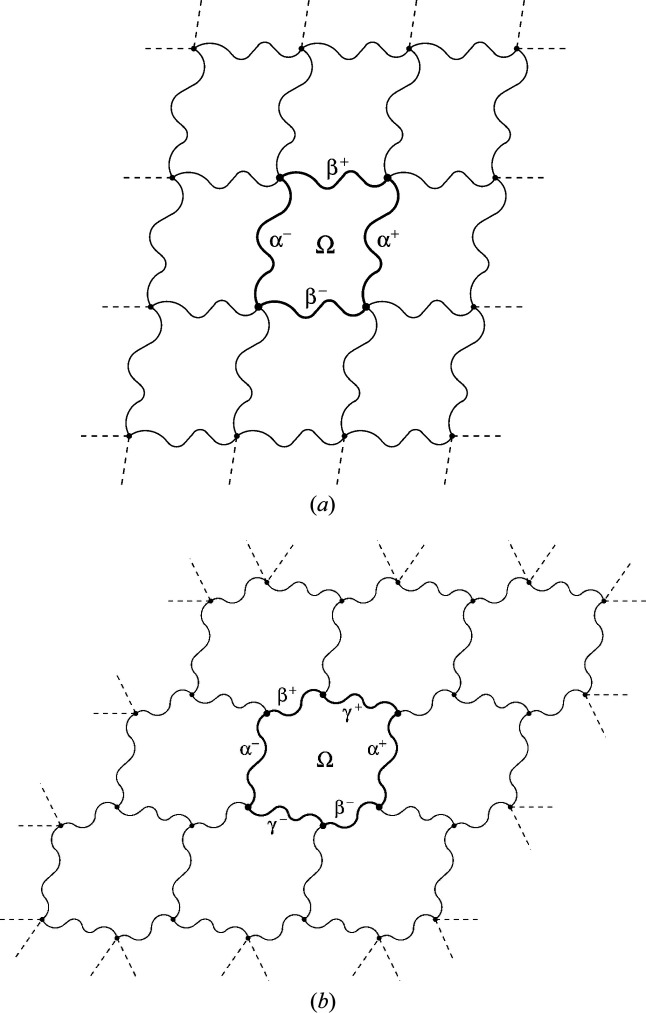
Each periodic tiling can be realized with a generalized quadrangle or hexagon. We distinguish the set of edges in both situations and divide them into two or three translation-equivalent pairs.

*Case I*. The boundary of Ω is a union of four connected 1-complexes: 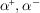, which are congruent via translation, and 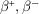, which are congruent as well [Fig. 3 ▸(*a*)].

*Case II*. The boundary of Ω is a union of six connected interlocking 1-complexes: 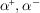, which are congruent via translation, and 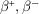 congruent as well, and finally 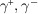 [Fig. 3 ▸(*b*)].

Lemma 5.1 Let 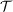 be a normal periodic tiling with generating region Ω. Let 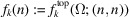 denote the number of *k*-cells in the finite tiling 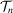. For a fixed 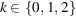 the function 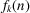 which counts the number of *k*-dimensional cells in 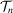 is a quadratic polynomial. Moreover, we have 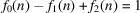
for every *n*.

**Proof**

The first claim was proved by Naskręcki *et al.* (2021*a* ▸). The second claim is a consequence of the fact that 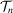 is a simply connected region (it is actually contractible to a point). Then the Euler characteristic of such a polygonal region is 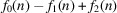 and equals 1 in that case [*cf*. Naskręcki *et al.* (2022 ▸) for such calculations].

□

By orbifolding the complex 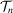 by the action of the group 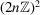 one obtains a CW complex 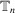 which is a 2D torus. We denote by 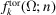 the number of *k*-cells in the torus 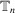.

Lemma 5.2 With the assumptions of Lemma 5.1[Statement lemma5.1] we have that there exists a triple of integers 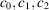 such that the vector 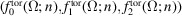 is a 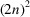 multiple of a constant vector 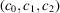.

**Proof**

The number 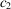 is equal to the number of 2-cells in Ω.

Case I. To compute 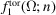 we observe that the 1-cells in the boundary of Ω are naturally divided into four subsets 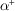, 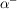, 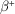 and 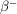 which get identified in the orbifolding process as a torus. Hence, the inner edges of Ω in 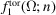 contribute with 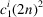 (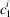 being the number of such inner edges in Ω) and the boundary edges contribute with 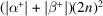. Since the Euler characteristic of the torus is zero, it follows that 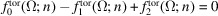; thus the polynomial 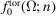 is of the form 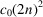 as well.

Case II. We modify the argument of case I by introducing the third pair of 1-complexes 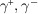. The modification happens in the contribution of the boundary edges, which is now 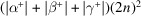.

□

To compute explicitly the numbers 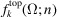 we study first the functions 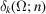 defined as 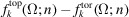.

Lemma 5.3 Let 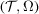 be as defined in Lemma 5.1[Statement lemma5.1]. We have 
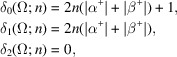
for case I. For case II we have
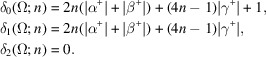

**Proof**

The correction 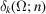 corresponds to the boundary contribution of the tiling 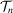. Hence the term for 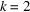 vanishes.

Case I. For 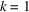 we observe that the boundary of 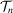 consists of 2*n* copies of elements in 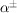 and in 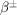. Since under the orbifolding to 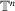 the elements from the 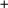 set are identified with the elements of the 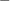 set, the formula follows.

Case II. In this case we have two boundaries of type 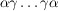, 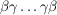 with 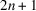 elements each and additionally one chain of type γ. These three chains are connected.

□

Let 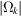 denote the cardinality of the set of *k*-cells of the CW complex Ω. Similarly, let 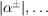 denote the number of 1-cells contained in 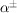
*etc*. Based on the Lemma 5.3[Statement lemma5.3], we obtain the following explicit formulas for the functions 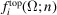.

Lemma 5.4 Let 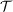 be a normal periodic tiling with the generating region Ω. We have the following formulas for the growth functions 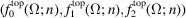 expressed in the graph radius *n*.

Case I polynomials: 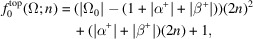

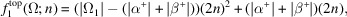

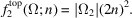

Case II polynomials: 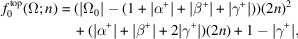

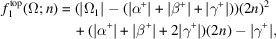

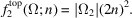

**Proof**

Since 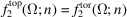 we have 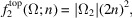
where 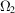 is the set of faces of Ω. Case I: we have 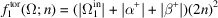
and 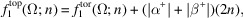
hence 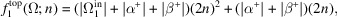
where 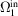 is the set of inner edges of Ω (not in the boundary). Finally, notice that 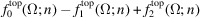 = 1 and 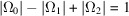 (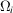 is the set of *i*-dimensional faces of Ω) and 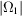 = 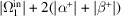, hence 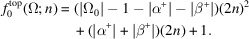
A similar proof applies to Case II.

□

In a periodic tiling one can independently change two parameters, say 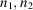. Thus, when the periodic tiling is given with the fundamental domain Ω, we can generalize the argument presented in Lemmas 5.1[Statement lemma5.1]–5.4[Statement lemma5.4] to this setup. As a result, we obtain the following formulas.

Theorem 5.5 Let 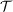 be a periodic normal tiling with the generating region Ω. For each positive 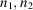, one gets 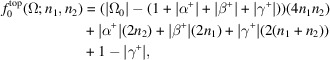

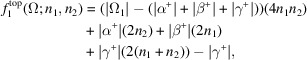

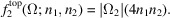

If we take into account the normalization of the variables discussed in Section 4.1, the formulas in Theorem 5.5[Statement theorem5.5] provide a way to compute the topological growth functions for periodic tilings that does not require any linear algebra computations. A numerical approach (which uses explicit matrix computations) is presented in Appendix *B* (Section *B*1). The importance of these formulas is theoretical. It shows that the coefficients are always integers and moreover we obtain a relation of the coefficients of the growth functions with the combinatorial data describing the fundamental domain Ω. For the tilings in Fig. 1 ▸ we have compiled in Table 1 ▸ a list of data attached to fundamental regions Ω that are indicated with thick black edges. The formulas from Theorem 5.5[Statement theorem5.5] together with Table 1 ▸ reproduce the normalized polynomials presented in Table 2 ▸.

**Figure 4:**
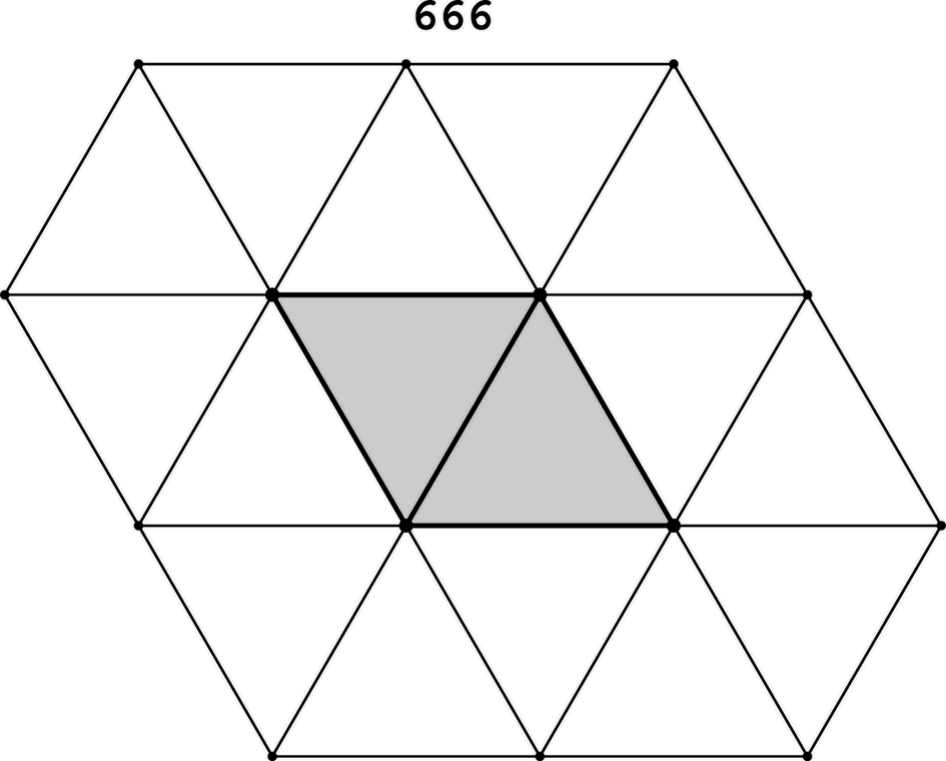
The 666 tiling (thin lines) presented as rhombic tiles (one tile shaded gray) having four edge-neighbors each. The rhombic tiles, each comprised of two adjacent 666 triangular tiles, correspond to the crystallographic unit cells of the hexagonal system.

Example 5.6 The 11 Laves tilings. Among the 11 types of tilings presented in Fig. 1 ▸, some tiles have four neighbors and have the shape of a square (4444 and 884) or a ‘wobbly’ square (43433), while others have six neighbors and the shape of a hexagon. However, certain tilings, shown in Fig. 1 ▸ as built from hexagons, can also be presented as built from rhombuses (666, 6434, 1264, 12123) or ‘wobbly’ rhombuses (63333). For example, the 666 case in Fig. 4 ▸ is an alternative of Fig. 1 ▸(*c*). Such rhombuses represent the proper crystallographic unit cells of the hexagonal system. Each rhombus has then four (edge) neighbors. In the remaining three tilings (333333, 44333, 6363) the individual tiles can only be presented as hexagons, and not as rhombuses. Of course, even for the hexagonal 333333 and 6363 tilings the crystallographic unit cells can be selected as rhombuses, but such cells would not have their vertices at the hexagonal lattice nodes. The 44333 tiling has rectangular symmetry and each alternatively selected tile always has six neighbors. The topological growth functions for the 44333 tiling and the other ten Laves tilings are presented in Table 2 ▸.

## 6. Crystallographic growth functions of a tiling

In this part, we will present a method to compute the topological and crystallographic growth functions that is based on linear algebra. The reason why these computational methods are useful stems from the fact that the identification of the boundary data for the domain Ω is not always easy and completely trivial. In comparison, the methods presented in Section *B*1 are straightforward to implement and very robust.

We fix a tiling 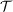 with the fundamental domain Ω. The translations in 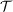 are spanned by two non-parallel vectors 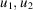 which form a parallelogram 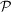. We fix a starting point 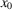 in Ω and consider, as discussed in Section 4.2, the set of three growth functions relative to 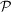 and its shifts. We are interested in finding a pattern for the growth functions relative to 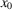. The main observation is that these functions vary with 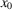 and are ‘locally constant’, *i.e.* they stay fixed when 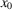 moves within the relative interior of each fixed cell. The precise conditions at the boundaries were described in Section 4.3.

### 6.1. Properties of the crystallographic growth functions

In particular, we can attach to such domains of invariant growth functions a given color. Each such domain is either a point, a line segment or a parallelogram *etc*. Such colored diagrams carry rich information about the tiling itself.

We compare two tilings which are topologically, but not geometrically, identical. The topological growth functions are the same for them but the crystallographic growth functions are distinct.

One can notice that the leading terms of the growth functions are identical in both situations, but the geometric pattern in which these triplets of functions appear is different.

The patterns satisfy further properties, namely:

(i) Invariance of the color-encoding scheme under the change of basis for the crystallographic lattice.

(ii) Compared with the topological functions, the crystallographic ones are sensitive to the deformations of the lattice, *i.e.* they encode point-group symmetries in the color arrangement (there is a finite group action on the arrangements of colorings of cells).

(iii) The crystallographic growth functions encode locally the same information as the topological growth functions but the extra dependence on the relation with the frame of reference provides an additional input about the symmetry of the group (the topological growth functions are completely insensitive to the difference between two topologically equivalent, yet geometrically different, tilings).

**Figure 5:**
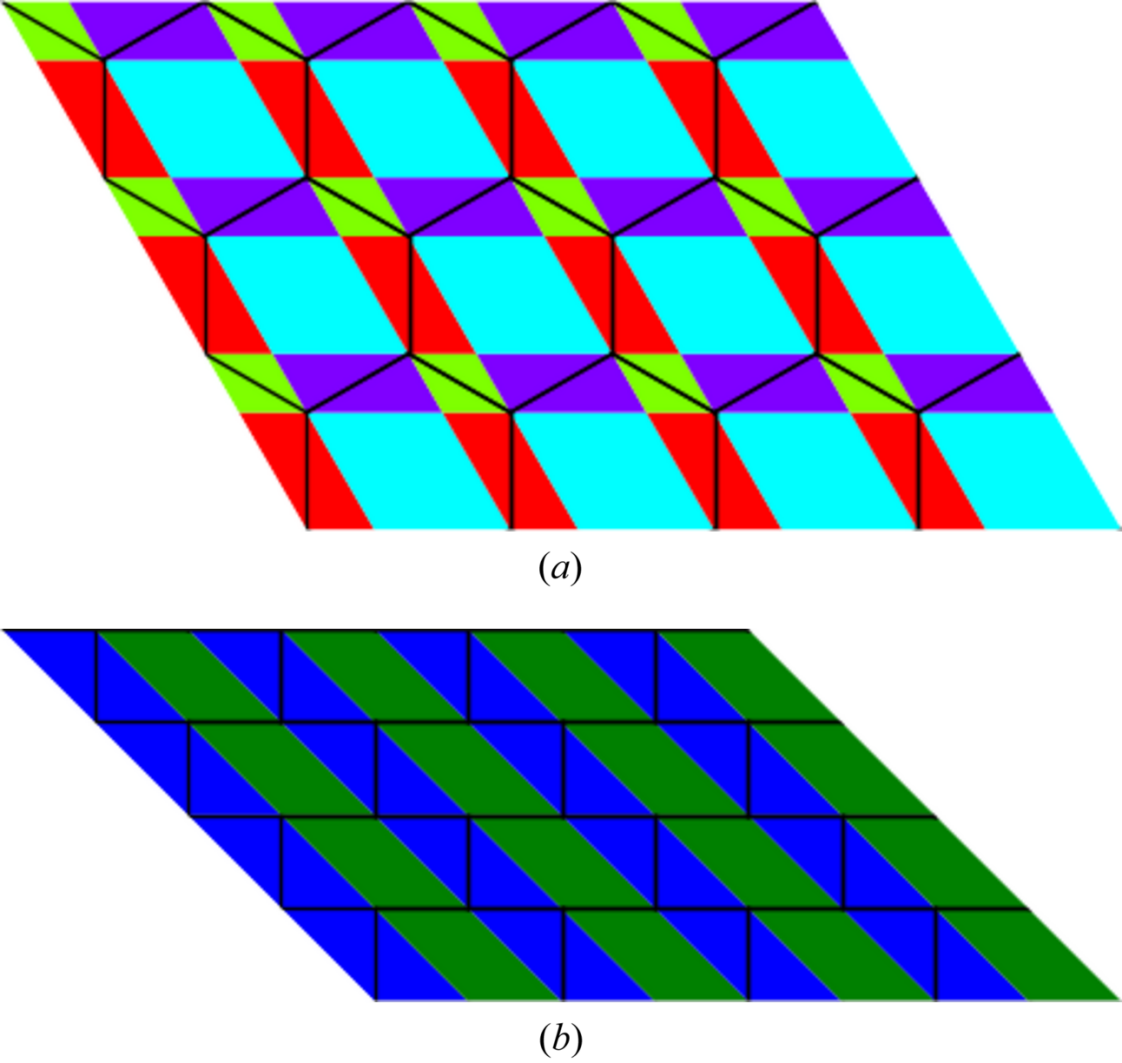
Diagram (*a*) represents the changes of the growth functions as we move across the plane tiled according to the topological 333333 pattern, with symmetry group *p*6*mm*. Diagram (*b*) has a tiling that is topologically equivalent but the symmetry group is reduced to *c*2*mm*. This is reflected in the color (growth functions) pattern.

We illustrate this idea using, as examples, the orphic diagrams in Figs. 5 ▸ and 6 ▸.

### 6.2. Orphic diagrams

We note that the diagrams color-coding the crystallographic growth functions bear striking similarity to the paintings of the orphists. We call, therefore, our diagrams orphic diagrams. Around 1912 a new style in art, called orphism, was born. We quote two descriptions of the orphic style.

‘The term, sometimes called orphic cubism, was coined around 1912–13 by the French poet and art critic Guillaume Apollinaire and used to distinguish their work from cubism generally. The name comes from the legendary ancient Greek poet and musician Orpheus. Its use by Apollinaire relates to the idea that painting should be like music, which was an important element in the development of abstract art. Robert Delaunay himself used the term simultanism to describe his work’ (https://www.tate.org.uk/).

Also

‘Orphist painters were interested in the geometric fragmentation of Cubism, but – unlike the Cubists, who removed almost all color from their paintings, and rather like the Fauvists – they considered color to be a powerful esthetic element’ (Encyclopedia Britannica, https://www.britannica.com/).

**Figure 7:**
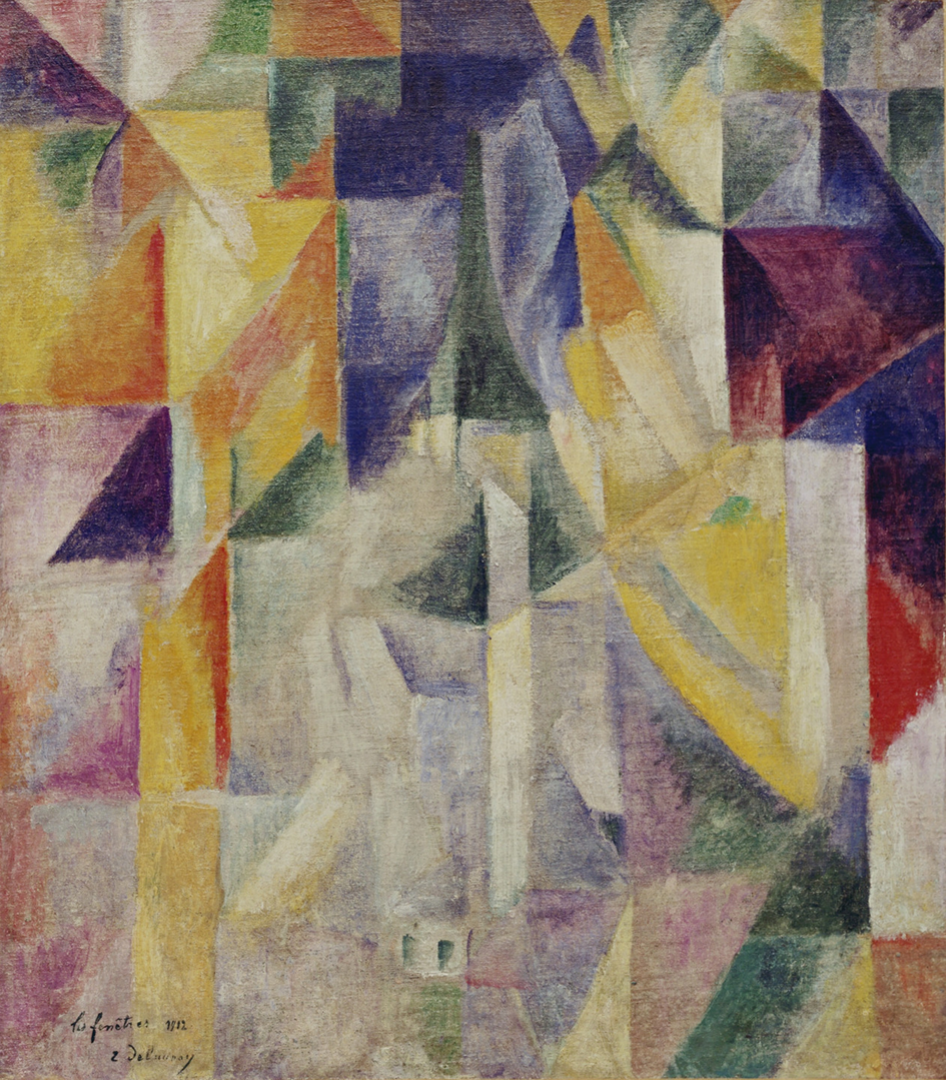
Robert Delaunay; Windows; Paris 1912; an example of orphic art.

The main orphists were Robert Delaunay (Fig. 7 ▸), his wife Sonia Terk Delaunay, and František Kupka.

More formally, we capture the ‘spirit’ of the orphic diagrams in the following definition.

Definition 6.1 (Orphic diagram) Let 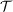 be a periodic normal tiling with a generating region Ω, translation vectors *u* and associated parallelogram 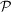. The associated crystallographic coloring 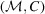 in the sense of Definition 4.12[Statement definition4.12] defines a function 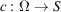
which is obtained as the restriction of 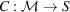 to the set Ω. The pair 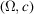 is called the *orphic diagram* of 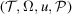.

In practice, the choice of the ‘colors’ in the set *S* is arbitrary. In the pictorial representation of the orphic diagram we try to choose highly contrasting colors for the neighboring regions. Also, when faced with the representation of the 0D, 1D and 2D regions we decided to show the 0D and 1D features in a separate picture (*cf*. Fig. 9). The color mappings for each case are independent.

Example 6.2 Reconstruction of point-group symmetry from its orphic diagram. In this example we investigate the orphic diagram associated with tiling 43433 from Fig. 1 ▸. The coefficients of the crystallographic growth functions are given in Table 3 ▸.

**Figure 8:**
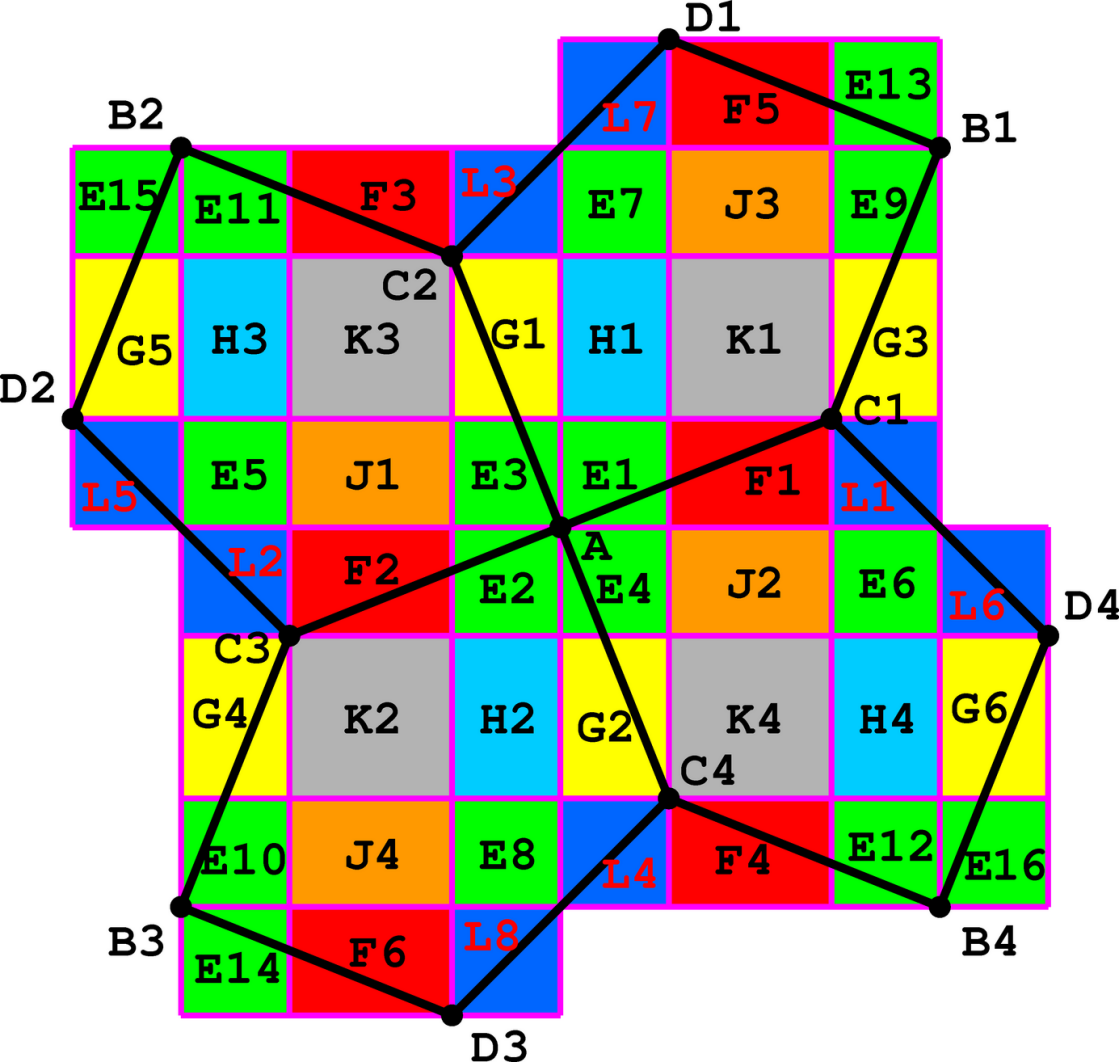
The orphic diagram generated for the 43433 tiling, in which each color represents a different triplet of growth functions. Each colored region (labeled by letters E to L) corresponds to growth functions obtained by assuming the origin of the crystallographic unit cell within that region. The letters A, B, C, D mark the vertices of the pentagonal tiles of the 43433 tiling. The pink lines mark the coordinates of the tile vertices in the *x* and *y* directions. Please note that the symmetry of this orphic diagram is *p*2*gg*. See the text for detailed explanations.

The orphic figure (Fig. 8 ▸) has interesting symmetry properties. For zero degrees of freedom (vertices), the figures are invariant (symmetric) with respect to 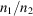 exchange. For one and two degrees of freedom, only some regions possess this symmetry.

There is a true vertical glide line *g* passing through the centers of squares K1 and K4 (as well as through K2 and K3, and through the centers of regions F5, J3, F1, J2, F4). It allows a right–left reflection with a simultaneous parallel translation by 1/2 of the unit-cell edge, after which operation the color pattern is the same. For example, H4 is transformed into H1, E6 into E7, L1 into L7 *etc*. There are also horizontal glide lines through the centers of square pairs K2/K4 and K1/K3. The vertical and horizontal *g* lines are shifted by 1/2 of the unit-cell edge along *y* and *x*, respectively. Note that the unit-cell corners are at B1, B2, B3 and B4. In addition, there are twofold rotations around the centers of the four green squares, *i.e.* at the unit-cell corners (points B) and at its center (point A), as well as at the common vertices of two navy-blue squares (*i.e.* at the center of the unit-cell edge). The color pattern has, therefore, the rectangular *p*2*gg* symmetry.

One notes, however, that the pattern has in fact apparent higher symmetry, that we term ‘pseudosymmetry’, which holds for the shapes of the orphic tiles but not for their color, and which is described as follows. There is a diagonal ‘pseudomirror line’ passing through the centers of squares L6, L1, K1 and E7, which transforms these squares onto themselves, and the adjacent pairs of regions of different colors onto one another, *e.g.* F1–G3 or H1–J3. These varicolored pairs correspond to polynomials that differ only by a swap of the 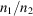 coefficients. It is a logical consequence of the fact that a diagonal inversion exchanges the horizontal and vertical directions. Analogous ‘pseudomirrors’ bisect squares K4 and K3, as well as E8, K2, L2 and L5; and in the perpendicular direction K2, K1 *etc*. Also points A and B1–B4 are the sites of ‘pseudofourfold’ axes, which exchange the polynomial coefficients within the square pairs H1/J2, H2/J2, F1/G2, F2/G1, as well as F5/G6, J3/H4 *etc*. Such a (pseudo)symmetry pattern corresponds to the square group *p*4*gm*, which is the space group of the original underlying 43433 tessellation of the plane with pentagons.

It is interesting that the navy-blue squares L (and their two edges) have ‘non-Eulerian’ polynomials, for which the telescoping sum of coefficients is not equal to 1, but 2.

**Figure 9:**
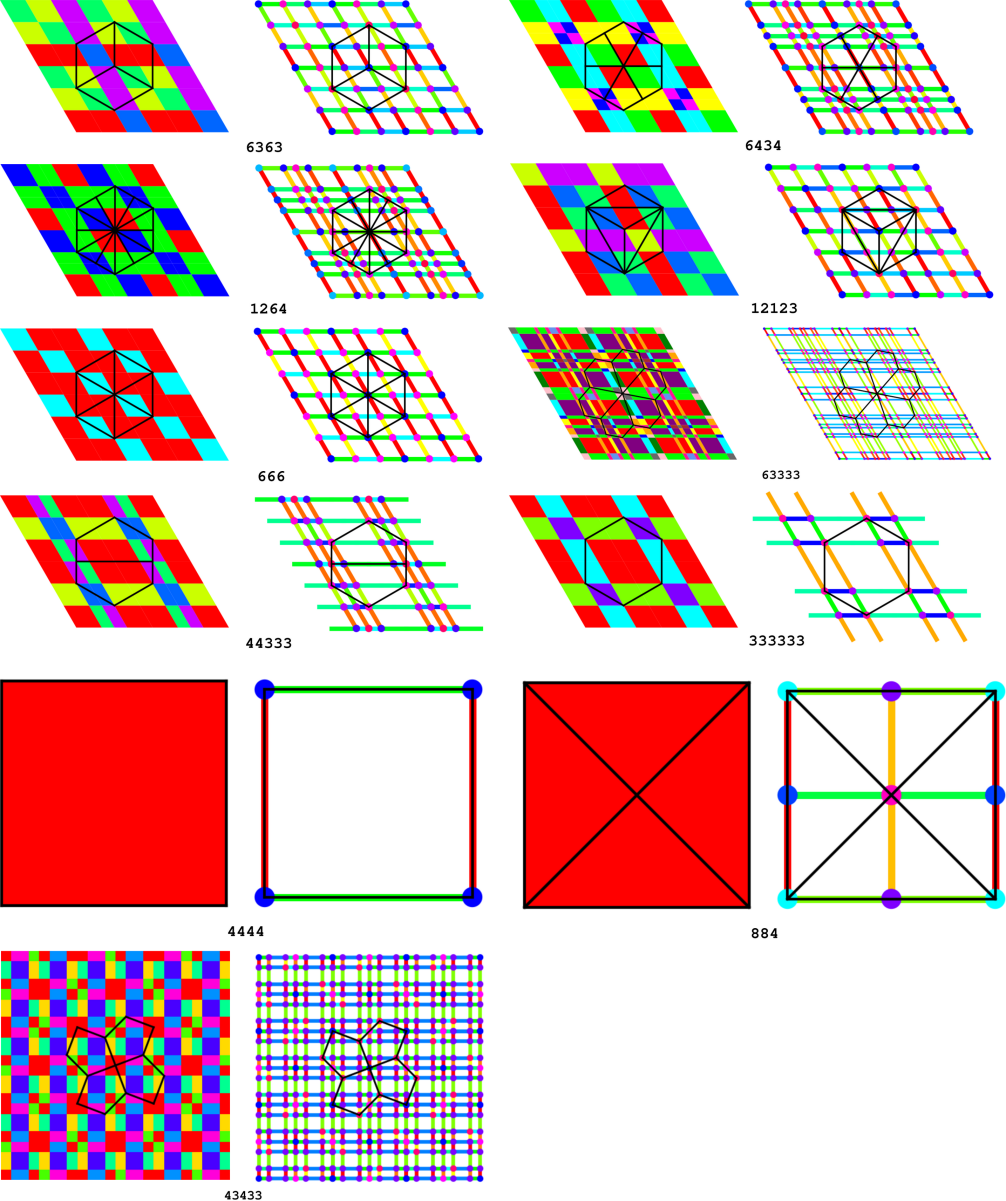
A pair of orphic diagrams generated for each tiling in Fig. 1 ▸. In each case, the left diagram corresponds to changes of the growth functions in 2D regions and the right diagram shows how they change at the specific boundaries. For each tiling type, a given color represents a unique triple of growth functions.

Example 6.3 Orphic diagrams of standard tilings, *cf*. Fig. 1 ▸. To illustrate the wealth of information contained in the orphic diagrams (which represent crystallographic growth functions), we tabulate them for the standard tilings presented in Fig. 1 ▸. Readers can find the precise polynomial functions describing each color in the supporting information files deposited in *Zenodo* (Malinowski, 2022 ▸). Each color represents a unique triple of polynomials, which correspond to the growth functions of the vertex set, edge set and face set, starting in the unicolored region. When the color changes, over a region boundary, we obtain a different triple of polynomial functions. We notice that the boundaries of the 2D unicolored regions typically have different growth functions, so in fact the orphic diagram contains information in its whole 2D structure as a CW complex. We record in Fig. 9 ▸ the orphic diagrams corresponding to the tilings of Fig. 1 ▸.

## 7. Applications and properties of the growth functions

The main reason for studying the growth functions of tessellations is the expectation that such algebraic–combinatorial data might be sufficient to encode the information about a tessellation. (We want to define enough numerical properties that allow us to reconstruct a tessellation up to isometry.) This expectation is expressed using graph theory in the book by Sunada (2013 ▸). While the growth functions are easy to compute, the reconstruction of the information about the tessellation from the functions themselves is a more challenging problem. In the following subsections we point to places where the growth functions connect to other interesting invariants of tessellations.

Different variants of the growth functions carry a wealth of information about the tessellations. In particular, we show how such information can be extracted and used.

### 7.1. Relation to the modified Euler characteristic

Given a periodic tiling 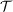, we consider its symmetry group *G*. The quotient space 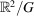 is a topological space which carries a natural structure of an orbifold 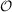 (*cf*. Naskręcki *et al.*, 2021*a* ▸, 2021*b* ▸). Computation of the associated Euler characteristic attached to the orbifold 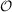 consists of computing a vector 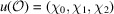 of rational numbers such that 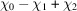 is the orbifold Euler characteristic of 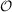 in the sense of Thurston (Naskręcki *et al.*, 2021*a* ▸). We proved that the vectors 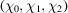 and 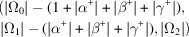
are proportional. The proportionality constant is computed from the quotient of the surfaces of Ω and the parallelogram 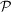 which is spanned by the translation subgroup vectors. The topological growth functions carry information about the orbifold 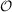. However, the topological growth functions also contain information about the combinatorics of the boundary of Ω.

### 7.2. Identification of a tiling using the growth functions

To show the practical utility of the growth functions, we carried out the following simulation. Starting with the tiling 884 generated in a sufficiently large range (at least four steps in each direction), we randomly (with probability 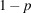) remove the central node of a square. Next, we generate the topological growth functions by starting the calculation at 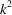 different points. The mean of the coefficients of the fixed-index polynomials is rounded to the nearest integer. We calculate for a given probability *p* how many times the tiling is classified as 884 or 4444 (or as ‘other’). The numerical results are summarized in Table 4 ▸. *k* represents the number of sampling points in each direction. In a column with fixed *k*, for each *p* we have three numbers, showing the number of polynomials that were recognized as the topological growth functions of tiling 884, tiling 4444 and unrecognized functions, respectively.

## 8. Outlook

In this paper we established some basic properties of the growth functions attached to 2D tilings. In Section 5 we showed that the leading terms of the growth functions form a vector that can be described in terms of the contributions to the modified Euler characteristic (orbifold characteristic). In Section 4 we proved several statements that describe the fundamental geometric properties of the crystallographic growth functions, encoded the properties of the tessellation in the crystallographic coloring functions, and explored the basic properties and applications of the coloring functions and orphic diagrams in Sections 6, 7.

We expect that it will be possible to find further properties of these functions. In particular, we think that it should be possible to find a relation between the polynomials that define the growth functions of dual tilings.

Is there a similar relation between the secondary terms of the growth functions and the properties of the region Ω? In particular, is there a relation (similar to isoperimetry) between the area and diameter of Ω? We plan to investigate these and other problems related to the tessellations of space as a continuation of this project, by means of convolutional neural networks and supervised machine learning. Among those other problems is the discovery of a precise relation between the symmetry group of a tessellation and the coefficients of the growth functions. We plan to implement this strategy following the general scheme proposed by Davies *et al.* (2021 ▸).

### 8.1. Higher dimensions

**Figure 10:**
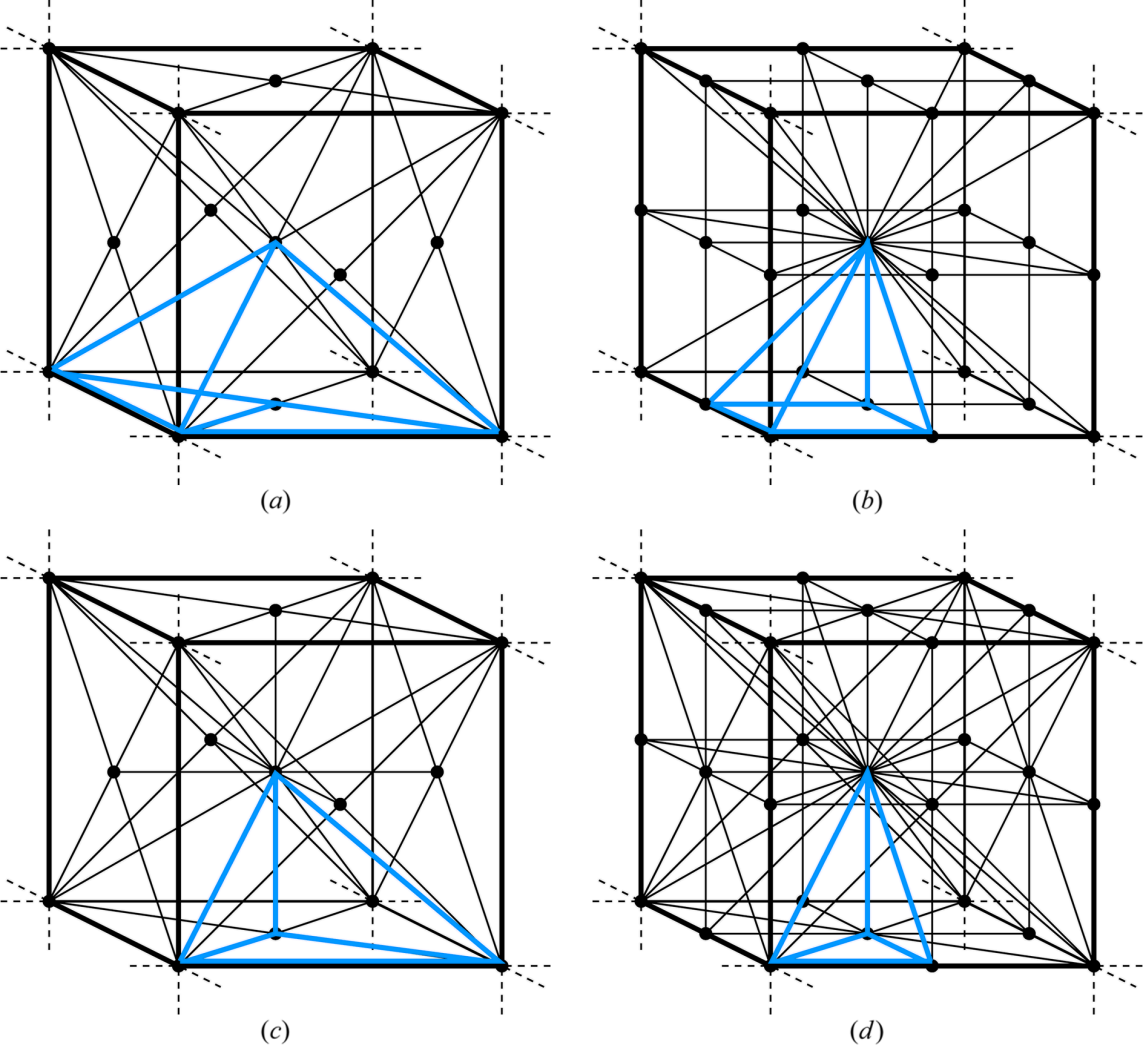
The unit cells of the primitive cubic symmorphic space groups, divided into individual asymmetric units, with one asymmetric unit highlighted in blue. (*a*) *P*23, (*b*) 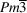, (*c*) *P*432 and 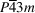, (*d*) 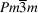.

**Figure 11:**
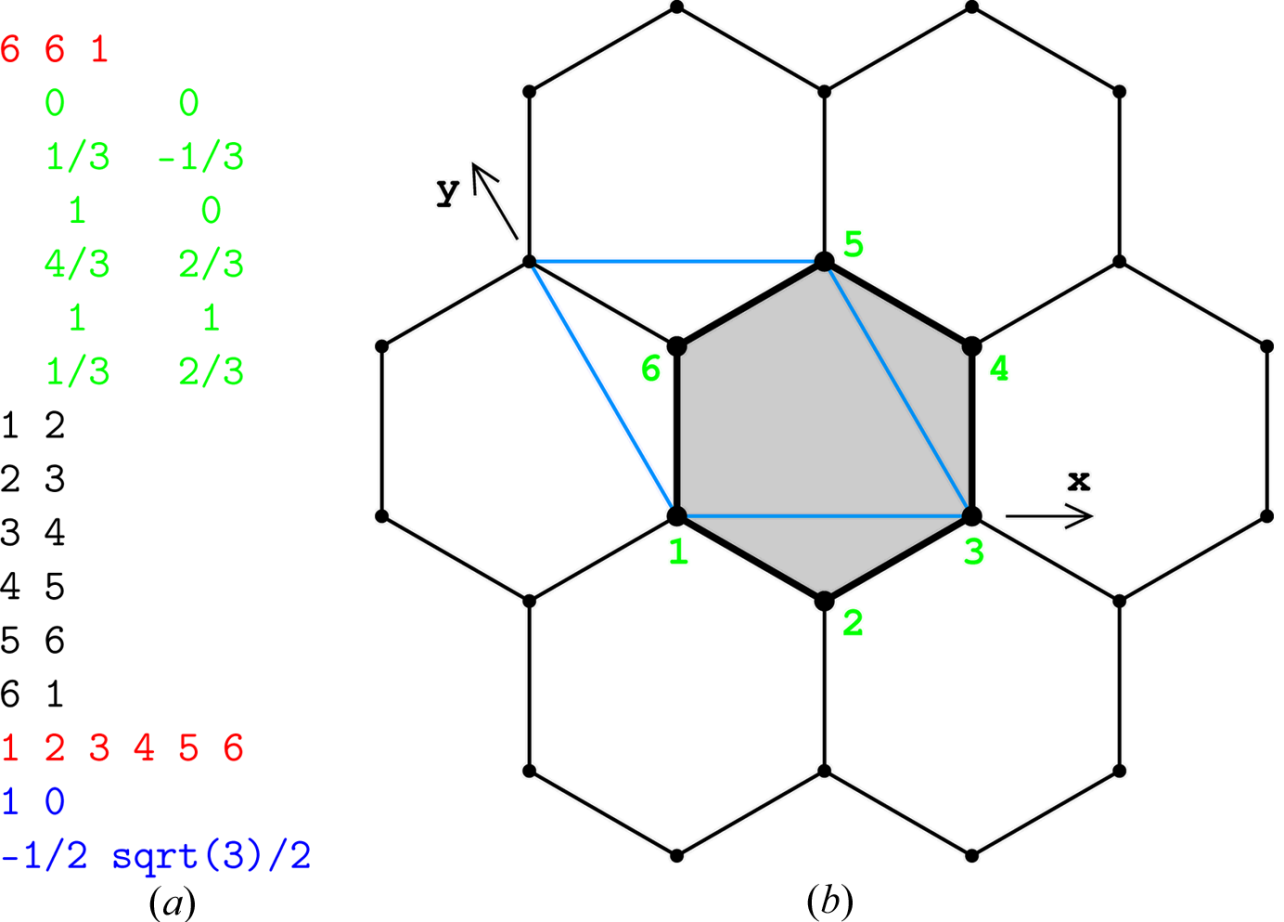
Sample input (*a*) encoding the 333333 tiling (*b*). The line 6 6 1 (red) gives 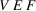, where *V* is the number of vertices, *E* is the number of edges and *F* is the number of faces. The next six 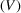 lines (green) describe the crystallographic coordinates of the vertices. The next six 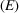 (black) lines describe the connectivity of the vertices (*i.e.* edges). Finally, the next one 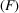 (red) line(s) describe the faces. The last two (blue) lines specify the translational lattice.

In 3D and higher dimensions of the Euclidean space, the growth functions become more complicated and their mutual relations are much harder to track. In addition, the number of different tessellations is unknown even in 3D (*cf*. Goodman *et al.*, 2018 ▸, Section 3.2, p. 74, p. 3). As a reconnaissance exercise, we have computed a handful of examples for some crystallographic 3D tessellations, and present them in Fig. 10 ▸ and Table 5 ▸.

## Figures and Tables

**Figure 6:**
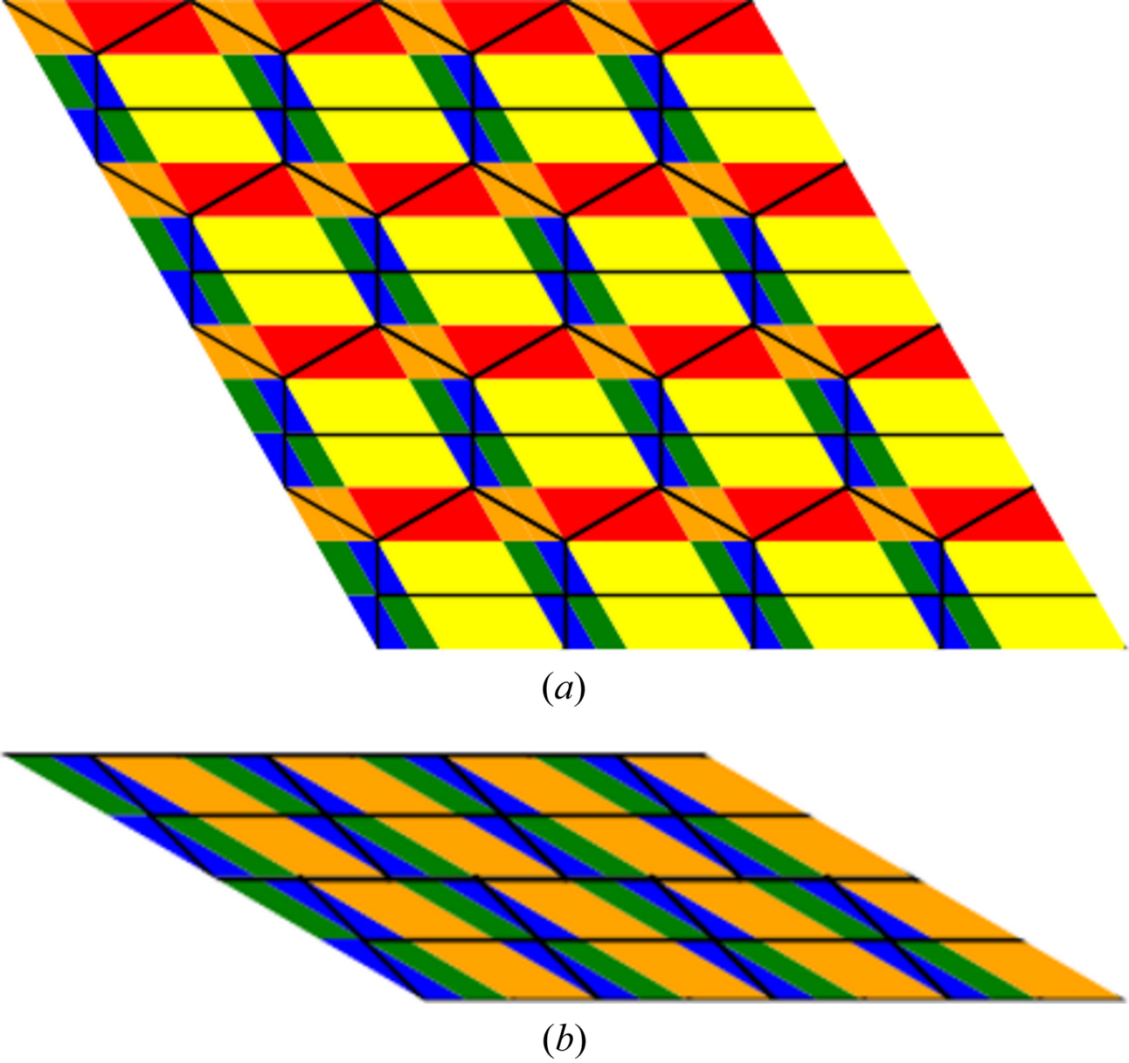
Diagram (*a*) represents the changes of the growth functions as we move across the plane tiled according to the topological 44333 pattern, with symmetry group *c*2*mm*. Diagram (*b*) has a tiling that is topologically equivalent but the symmetry group is reduced to *p*2. This is reflected in the color (growth functions) pattern.

**Table 1:** Numbers 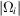 are counts of *i*-dimensional cells of the fundamental region of Ω attached to a tiling of the type described in Fig. 1 ▸ The numbers 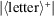 count the edges of a given type.

Tiling	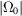	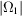	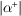	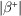	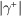	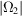
4444	4	4	1	1	0	1
333333	6	6	1	1	1	1
666	7	12	1	1	1	6
884	5	8	1	1	0	4
44333	8	9	2	1	1	2
43433	13	16	3	3	0	4
6363	7	9	1	1	1	3
6434	13	18	2	2	2	6
1264	13	24	2	2	2	12
12123	7	12	1	1	1	6
63333	19	24	3	3	3	6

**Table 2:** Topological growth functions for the polygonal units filling the plane by translations, corresponding to the 11 types of tilings shown in Fig. 1 ▸ Each row represents three functions, for the growth of the number of vertices (V), edges (E) and faces (F). Each function is normalized, *e.g.* the tiling of type 4444 from Fig. 1 ▸ has the growth functions 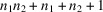, 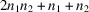 and 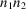.

		V				E				F			
Type	Symmetry	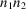	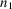	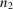	1	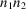	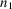	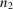	1	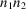	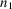	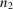	1
4444	*p*4*mm*	1	1	1	1	2	1	1	0	1	0	0	0
333333	*p*6*mm*	2	2	2	0	3	2	2	−1	1	0	0	0
666	*p*6*mm*	3	2	2	0	9	2	2	−1	6	0	0	0
884	*p*4*mm*	2	1	1	1	6	1	1	0	4	0	0	0
44333	*c*2*mm*	3	2	3	0	5	2	3	−1	2	0	0	0
43433	*p*4*gm*	6	3	3	1	10	3	3	0	4	0	0	0
6363	*p*6*mm*	3	2	2	0	6	2	2	−1	3	0	0	0
6434	*p*6*mm*	6	4	4	−1	12	4	4	−2	6	0	0	0
1264	*p*6*mm*	6	4	4	−1	18	4	4	−2	12	0	0	0
12123	*p*6*mm*	3	2	2	0	9	2	2	−1	6	0	0	0
63333	*p*6	9	6	6	−2	15	6	6	−3	6	0	0	0

**Table:** (*a*) Coefficients of the growth functions for the 2D regions (their interiors) in Fig. 8 ▸. Each row represents three functions, for the growth of the number of vertices (V), edges (E) and faces (F). Each function is normalized, *e.g.* the region E has the growth functions 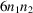, 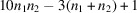, 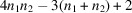.

	V				E				F			
	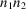	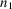	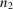	1	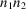	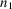	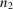	1	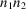	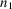	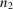	1
E	6	0	0	0	10	−3	−3	1	4	−3	−3	2
L	6	0	0	0	10	−3	−3	1	4	−3	−3	3
J	6	0	0	0	10	−3	−2	0	4	−3	−2	1
F	6	0	0	0	10	−3	−2	1	4	−3	−2	2
H	6	0	0	0	10	−2	−3	0	4	−2	−3	1
G	6	0	0	0	10	−2	−3	1	4	−2	−3	2
K	6	0	0	0	10	−2	−2	0	4	−2	−2	1

**Table:** (*b*) Coefficients of the growth functions for the interiors of the line segments in Fig. 8 ▸. Each row represents three functions, for the growth of the number of vertices (V), edges (E) and faces (F). The syntax XYh/v indicates the interior of the edge between regions X and Y (v stands for vertical, h for horizontal edge).

	V				E				F			
	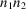	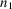	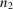	1	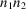	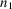	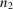	1	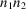	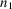	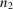	1
EEv, EJv	6	0	1	0	10	−3	−1	0	4	−3	−2	1
ELv, EFv	6	0	1	0	10	−3	−1	1	4	−3	−2	2
FLv	6	0	1	0	10	−3	−1	0	4	−3	−2	2
GHv, GKv, HKv	6	0	1	0	10	−2	−1	0	4	−2	−2	1
EEh, EHh	6	1	0	0	10	−1	−3	0	4	−2	−3	1
EGh, ELh	6	1	0	0	10	−1	−3	1	4	−2	−3	2
GLh	6	1	0	0	10	−1	−3	0	4	−2	−3	2
FJh, JKh, FKh	6	1	0	0	10	−1	−2	0	4	−2	−2	1

**Table:** (*c*) Coefficients of the growth functions for the vertices in Fig. 8 ▸. Each row represents three functions, for the growth of the number of vertices (V), edges (E) and faces (F). The syntax XYZW indicates the vertex at the intersection of the 2D regions X, Y, Z and W.

	V				E				F			
	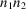	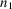	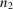	1	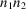	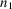	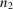	1	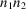	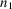	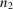	1
EEEE	6	1	1	1	10	−1	−1	0	4	−2	−2	0
EEGH, EGHL, EEFJ, EGJK, EFJL, EHJK, EFHK	6	1	1	0	10	−1	−1	0	4	−2	−2	1
EELL	6	1	1	0	10	−1	−1	1	4	−2	−2	2
FGKL	6	1	1	1	10	−1	−1	0	4	−2	−2	1

**Table 4:** The ability of the growth functions to recognize the tiling, when 884 is distorted towards 4444 1 − *p* is the probability of a removal of the central vertex (change from the 884 to 4444 tiling). *k* represents the number of sampling points in each direction. The three numbers in each row and column represent the numbers of generated polynomials that either are of type 884, of type 4444 or something else (*cf*. Section 7.2).

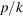	1			2			3			4		
0.01	0	962	38	0	995	5	0	1000	0	0	1000	0
0.1	0	672	328	18	826	156	0	984	16	0	997	3
0.2	4	406	590	91	553	356	13	915	72	7	977	16
0.5	53	55	892	399	124	477	434	410	156	582	375	43
0.8	424	1	575	672	0	328	918	11	71	986	0	14
0.9	663	0	337	847	0	153	988	0	12	998	0	2
0.99	955	0	45	997	0	3	1000	0	0	1000	0	0

**Table 5:** Crystallographic growth functions (of single variable *n*) of all elements of the unit cells shown in Fig. 10 ▸

Space group	V	E	F	I
*P*23	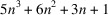	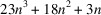	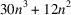	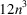
*Pm* 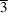	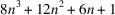	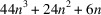	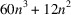	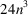
*P*432 and 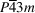	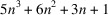	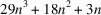	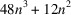	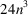
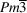 m	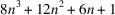	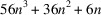	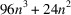	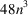

## Appendix A Mathematical glossary

This is a glossary of the less familiar mathematical terms, ordered alphabetically. In the text, after the first use of a glossary term, we include a cross-reference to this appendix ‘(Appendix *A*)’.

An *Archimedean tiling* is a tiling where every vertex has the same valency. Its dual is called the *Laves tiling*.

A *fundamental domain* is a shape that has the property that the space group maps every point in the Euclidean space to a unique point in the fundamental domain via a unique symmetry group operation.

A *CW complex* is a way of building spaces by starting with a collection of points and attaching cells of different dimensions to these points.

Think of a cell as a simple shape, like a line segment, a triangle or a cube. You start by taking a collection of points, and then you attach 1D cells (line segments) between them to form a network of connected line segments. Then, you can attach 2D cells (triangles or squares) to these line segments to form a more complex shape. Finally, you can attach 3D cells (cubes) to build a solid object.

In this way, you can construct a space by attaching cells of different dimensions to a collection of points, and this is called a CW complex, according to Whitehead (1949 ▸). The name CW stands for closure-finite weak topology. The cells can be attached in many different ways to create a wide variety of shapes and spaces, and this makes CW complexes a useful tool for understanding topological spaces.

In particular, a *k-cell* is a *k*-dimensional ball which we use as a building block in constructing CW complexes.

The *Euler characteristic* of a finite CW complex is the alternating sum , where  is the number of *k*-dimensional cells in the complex.

An *orbifold* is a topological space that is similar to a manifold, which is modeled locally with Euclidean space . Orbifolds differ from manifolds at the so-called singular subset. These singular points reflect the fact that orbifolds are obtained by the process of taking a quotient space of the Euclidean space by finite group actions. The singular points correspond to points in the Euclidean space that are stabilized by the group action.

An *orphic diagram* is a pictorial representation of the crystallographic growth functions attached to a fixed normal periodic tiling. Since the crystallographic growth functions depend on the initial vertex of the tiling, we associate with every point on the 2D plane an identical color if the triple of growth functions (for the vertices, edges and faces) stays the same. This provides a coloring of the plane with finitely many colors. More generally, orphic diagrams may be used to illustrate by color certain mathematical properties of certain  areas of the 2D plane.

An *(ordered) tuple* of elements  is a mathematical object that stores elements  with a strict ordering from left to right. It is modeled set-theoretically, *i.e.* by the construction of Kuratowski (1921 ▸), namely the 2-tuple  is the set  and the *N*-tuple is generated inductively.

In relative approach, given a CW complex structure on , one can consider a *family of growth functions* which depend on another tessellation or metric and which allow us to generate an index set  and a family  of finite CW complexes such that . In fact, we consider this sum as an inductive limit of finite CW complexes. For such a family we consider functions  which ‘measure’ the ‘growth’ of the tessellation  against the family . We consider  as a partially ordered set (Rasiowa, 1973 ▸, ch. IX), and consider the monotonic properties of the function *f* against such order.

## Appendix B Python code and algorithm

In the framework of this project we have developed a Python computer code that helps to illustrate the character of our theoretical results. In Section *B*1 we describe the algorithm for the calculation of the topological growth functions for arbitrary periodic tilings. The code is written in such a way that it can be easily adapted to any higher-dimensional case. In the supporting information files there is a loadable Python library that accepts as input a text file that describes completely the tiling of interest. An example input file encoding the 333333 tiling is presented in Fig. 11 ▸. The first line shows the number of cells in each dimension sorted in ascending order with respect to the cell dimension. The following lines encode the coordinates of the tiling vertices. The coordinates can be given using crystallographic or Cartesian convention. When describing vertices, the standard mathematical syntax can be used. Higher-dimensional cells are described after the vertices. Each line encodes one cell. Higher-dimensional cells are encoded with vertex indices. The last two lines contain information about translation vectors.

In Section *B*1 we discuss the details of the computation of the crystallographic growth functions. The theoretical foundations were discussed in Section 4.3.

### b1. Finding the topological growth functions

The algorithm get_topological_growth_polynomials finds the growth functions based on a list of 0D cells of the repeating unit, the number of 2D cells of the repeating unit and the translation vectors. The function  is of the following form:

The algorithm finds the coefficients  by solving the system of linear equations (1):

The values of the function  in (1) are found using the method get_k_cells_num described by Algorithm 1. The algorithm get_topological_growth_polynomials calculates the formula for function  using the identity  = . This identity is a simple consequence of the Euler characteristics for a simply connected CW complex.

### b2. Finding the crystallographic growth functions

The method denoted get_crystallographic_growth_functions finds the crystallographic growth functions (let us denote them ) based on a list of 0D, 1D and 2D cells of the repeating unit, the generating period vectors, a starting point, and two rational numbers to scale the frame.

The polynomials defining the growth functions for particular groups of arguments are found using an algorithm analogous to that of get_topological_growth_function used to find the function . Our algorithm for finding the crystallographic growth functions of a single variable is presented in Algorithm 2:

The algorithm for multivariable functions is analogous[Chem scheme2].

As in the case of finding the topological growth functions, also in this case we use information about the values of the growth function for given sets of arguments to find the polynomial coefficients. The required values are computed by the subroutine get_k_cells_num_parallelogram, which counts the numbers of *k*-cells of  that are completely contained within the parallelogram.

### b3. Computer code availability

The Python computer code developed to generate the growth functions discussed in this paper is freely available at the DOI link given by Malinowski (2022 ▸). The input consists of a text string entered at a prompt. The output consists of the explicit polynomial growth functions and their illustrations by orphic diagrams.
